# *Bacteroides fragilis* aggravates high-fat diet-induced non-alcoholic fatty liver disease by regulating lipid metabolism and remodeling gut microbiota

**DOI:** 10.1128/spectrum.03393-23

**Published:** 2024-02-27

**Authors:** Yumei Huang, Jiali Cao, Mengpei Zhu, Ziwen Wang, Ze Jin, Zhifan Xiong

**Affiliations:** 1 Department of Gastroenterology, Liyuan Hospital, Tongji Medical College, Huazhong University of Science and Technology, Wuhan, Hubei, China; National Health Research Institutes, Miaoli, Taiwan

**Keywords:** commensal *Bacteroides fragilis*, gut microbiota, non-alcoholic fatty liver disease, high-fat diet, lipid metabolism, short-chain fatty acids

## Abstract

Gut microbiota dysbiosis is a prominent determinant that significantly contributes to the disruption of lipid metabolism. Consequently, it is essential to the occurrence and development of non-alcoholic fatty liver disease (NAFLD). Nevertheless, the connection between diet and symbiotic gut microbiota in the progression of NAFLD remains uncertain. The purpose of this study was to explore the role of supplementing commensal *Bacteroides fragilis* (*B. fragilis*) on lipid metabolism, gut microbiota, and metabolites in high-fat diet (HFD)-fed mice, elucidating the impact of gut microbiota and metabolites on the development of NAFLD. Our study revealed that supplementation with *B. fragilis* exacerbated both weight gain and obesity in mice. *B. fragilis* exacerbated blood glucose levels and liver dysfunction in mice. Furthermore, an increase in liver lipid accumulation and the upregulation of genes correlated with lipid metabolism were observed in mice. Under an HFD, supplementation of commensal *B. fragilis* resulted in alterations in the gut microbiota, notably a significant increase in *Desulfovibrionaceae*, which led to elevated endotoxin levels and thereby influenced the progression of NAFLD. It was interesting that the simultaneous examination of gut microbiota metabolites revealed a more pronounced impact of diet on short-chain fatty acids. This study represented the pioneering investigation into the impact of *B. fragilis* on NAFLD. Our findings demonstrated that *B. fragilis* induced dysregulation in the intestinal microbiota, leading to elevated levels of lipopolysaccharide and dysfunction in glucose and lipid metabolism, thereby exacerbating NAFLD.

**IMPORTANCE:**

Some intestinal symbiotic microbes are involved in the occurrence of the metabolic disorders. Our study investigated the impact of supplementing commensal *Bacteroides fragilis* on host metabolism in high-fat diet-fed mice. Research results indicated that adding a specific bacterial strain to the complex intestinal microecology can worsen metabolic conditions. This effect mainly affects the structural diversity of intestinal microorganisms, the increase in harmful bacteria in the gut, and the elevation of endotoxin levels, blood glucose, and lipid metabolism, thereby impacting the progression of non-alcoholic fatty liver disease (NAFLD). Understanding the principles that govern the establishment of microbial communities comprising multiple species is crucial for preventing or repairing dysfunctions in these communities, thereby enhancing host health and facilitating disease treatment. This study demonstrated that gut microbiota dysbiosis could contribute to metabolic dysfunction and provides new insights into how to promote gut microbiota in the prevention and therapy of NAFLD.

## INTRODUCTION

The composition and functionality of gut microbiota can be influenced by host and dietary factors (1). The imbalance between the commensal and pathogenic microbiota causes dysbiosis of the gastrointestinal microbiota (2, 3). The important role of gut microbiota in the pathogenesis of non-alcoholic fatty liver disease (NAFLD) has recently been realized. However, they can also serve as reservoirs for pathogens and produce bioactive molecules that contribute to the pathogenesis of hepatic steatosis (4). Evidence from animal studies indicated that gut microbiota may play a pathogenic role in causing the occurrence of NAFLD (5, 6). Recent evidence has demonstrated the essential role that intestinal dysfunction played in the pathophysiology and etiology of metabolic disorders like type 2 diabetes (T2D), obesity, and NAFLD (7). Gut dysbiosis and alterations in metabolism of gut microbiota have been connected with the severity of NAFLD (8, 9). Zhuge et al. showed that Bacteroides were independently associated with non-alcoholic steatohepatitis (NASH), while *Ruminococcus* was significantly linked to fibrosis (6). Therefore, it is necessary to further understand the role of commensal gut microbiota in NAFLD.

*Bacteroides fragilis* (*B. fragilis*) is a Gram-negative obligate anaerobe that commensally lives in the lower gastrointestinal tract of mammals and exerts profound effects on host susceptibility to inflammatory disorders (10). Previous studies assigned *B. fragilis* strains by two subtypes: non-toxigenic *B. fragilis* (NTBF) strains that cannot produce or release *B. fragilis* toxin (BFT) and enterotoxigenic *B. fragilis* (ETBF) strains that carry BFT genes encoding *B. fragilis* toxin within the bacteria’s pathogenicity islands (BfPAI) (11–13). NTBF was reported to enhance the development of mucosal immunity and exert a suppressive effect on colitis, lupus nephritis, and viral encephalitis (14–16). Several studies have recently suggested that *B. fragilis* might have an increasingly significant pathogenic role in metabolic disorders such as diabetes, atherosclerosis, and obesity, demonstrating a causal association between the *Enterobacteriaceae* and metabolic disorder pathophysiology (17–20). Nevertheless, the exact role of NTBF in NAFLD remains uncertain.

Although the role of commensal intestinal *B. fragilis* in immune system development has been highlighted by animal and *in vitro* studies, there is a lack of research on the involvement of commensal *B. fragilis* in metabolic diseases (21). This study represented the first investigation into the impact of *B. fragilis* on the microbiome composition in NAFLD. We hypothesized that *B. fragilis* could cause the change of gut microbiota, influencing the occurrence of NAFLD. To more accurately replicate clinical circumstances, we first used an HFD-induced NAFLD model to study the effect of *B. fragilis* on NAFLD and its potential mechanism. We demonstrated that *B. fragilis* aggravated HFD-induced NAFLD by regulating lipid metabolism and remodeling gut microbiota.

## RESULTS

### Commensal *B. fragilis* triggered an increase in body weight and obesity in HFD-fed mice

**Fig 1:**
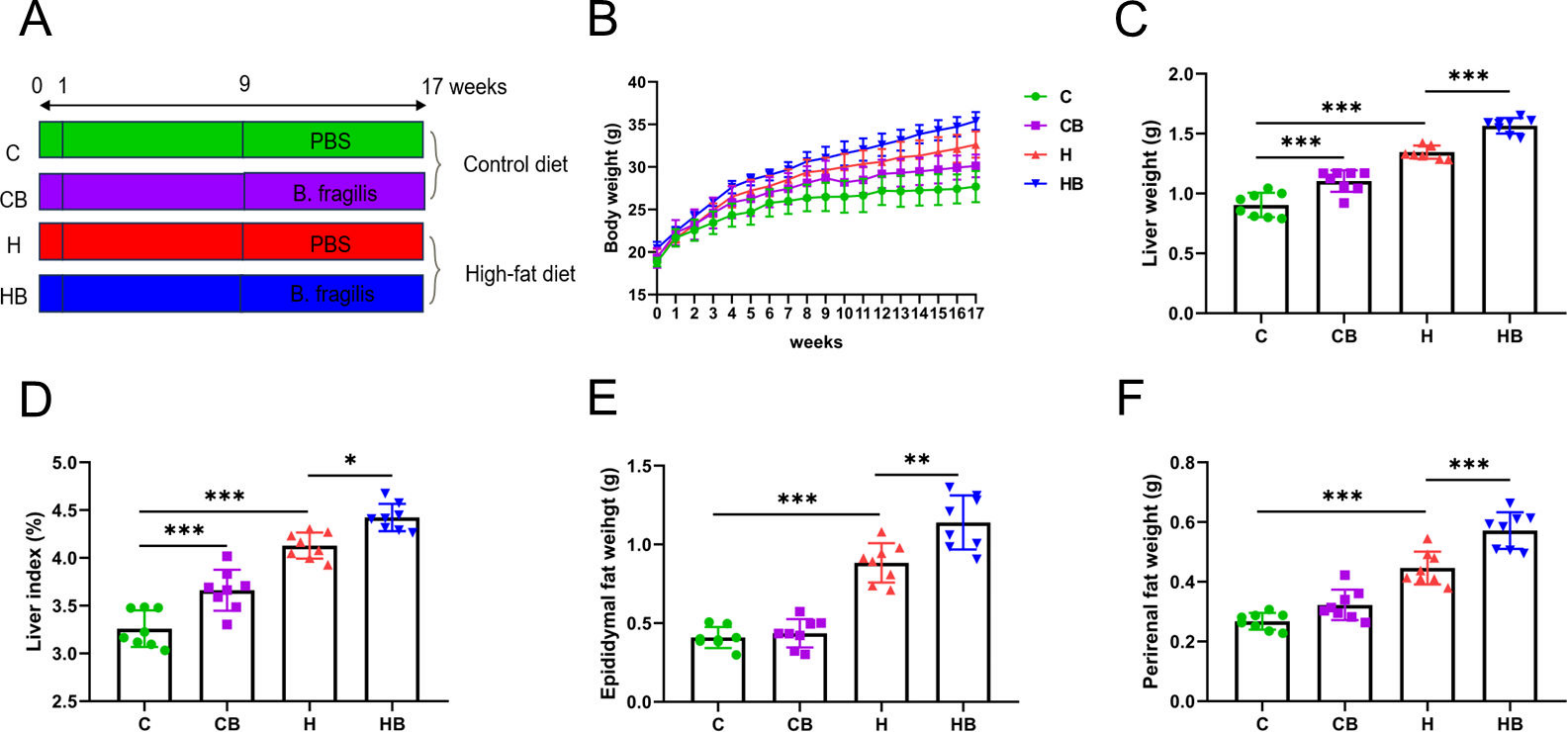
Commensal *B. fragilis* triggered an increase in body weight and obesity in HFD-fed mice. (**A**) Experimental protocol. (**B**) Body weight. (**C**) Liver weight. (**D**) Liver index. (**E**) Epididymal fat weight. (**F**) Perirenal fat weight. Values were shown as the mean ± SD (*n* = 8). Statistical analysis involved a two-way analysis of variance (ANOVA) followed by Tukey’s multiple comparisons test (**B**) and a one-way ANOVA followed by Tukey’s multiple comparisons test (C−F). *P < 0.05, **P < 0.01, and ***P < 0.001.

After being given 1 week to adjust to their circumstances, the experimental mice were subjected to an HFD for 16 weeks in order to induce liver steatosis. To investigate the effects of *B. fragilis* on NAFLD, we assigned four groups of mice: the C group received a chow diet with phosphate buffer solution (PBS), the CB group received a chow diet with *B. fragilis*, the H group received an HFD with PBS, and the HB group received an HFD with *B. fragilis* for the last 8 weeks (Fig. 1A). Mice fed an HFD developed obesity-related symptoms, as evidenced by an increase in body weight in comparison to mice on a control diet. Moreover, *B. fragilis* further exacerbated HFD-induced obesity (Fig. 1B). The weight growth in the H group began to significantly exceed that of the C group starting from the 5th week (*P* < 0.05). The weight growth in the HB group was obviously higher compared with that observed in the H group, starting from the 12th week (*P* < 0.05). At 17 weeks, in comparison to the C group, the H group demonstrated significant increases in liver weight (1.346 vs 0.9047), liver index (4.129 vs 3.259), epididymal fat (0.8837 vs 0.4088), perirenal fat (0.4462 vs 0.2676), and kidney (0.4010 vs 0.3327) (*P* < 0.01; Fig. 1C through F; Fig. S1A). Meanwhile, supplementation of *B. fragilis* further exacerbated the weight of these tissues in HFD-fed mice (*P* < 0.05).

### Commensal *B*. *fragilis* aggravated glucolipid metabolism and liver dysfunction in HFD-fed mice

**Fig 2:**
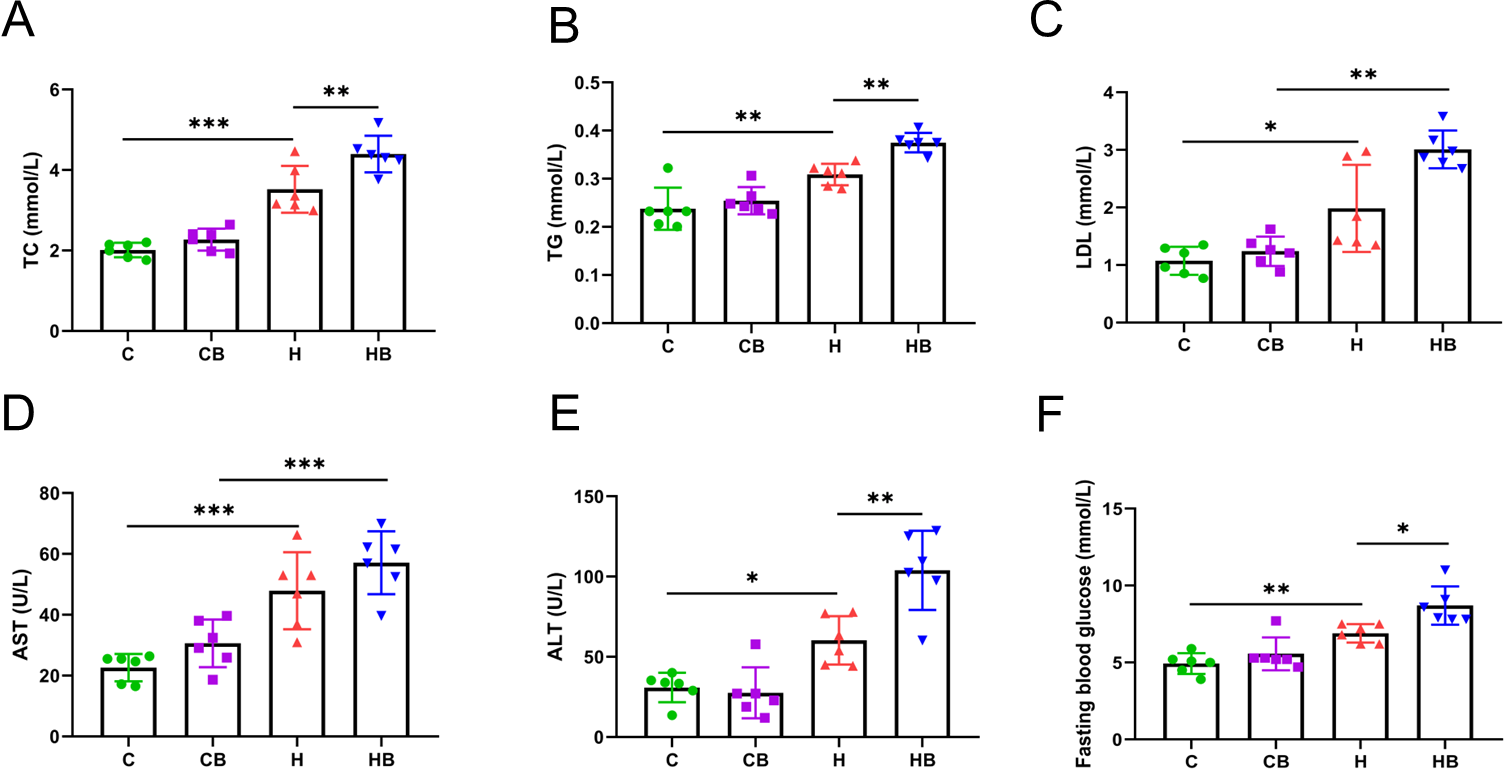
Commensal *B. fragilis* aggravated glucolipid metabolism and liver dysfunction in HFD-fed mice. (**A**) TC levels in the serum. (**B**) TG levels in the serum. (**C**) LDL-C levels in the serum. (**D**) AST levels in the serum. (**E**) ALT levels in the serum. (**F**) FBG levels. Values were shown as the mean ± SD (*n* = 6). Statistical analysis involved a one-way ANOVA followed by the Tukey’s multiple comparisons test. *P < 0.05, **P < 0.01, and ***P < 0.001.

The liver, being the principal organ responsible for glucolipid metabolism, exhibits impaired functionality as a significant abnormality. The mice in the H group had a more severe lipid metabolic problem than the C group, as shown by significantly higher serum total cholesterol (TC) (3.520 vs 2.012), triglyceride (TG) (0.3089 vs 0.2376), and low-density lipoprotein cholesterol (LDL-C) levels (1.985 vs 1.075) (*P* < 0.05; Fig. 2A through C). In comparison to the H group, the HB group showed elevated levels of serum TC (4.399 vs 3.520), TG (0.3749 vs 0.3089), and LDL-C (3.009 vs 1.985) levels in mice (*P* < 0.01). Although high-density lipoprotein cholesterol (HDL-C) levels in the H group were significantly lower than those in the C group, there was no statistically significant difference (Fig. S1B). Mice fed with an HFD exhibited exacerbated liver dysfunction, as evidenced by a significant increase in serum levels of aspartate aminotransferase (AST) (47.90 vs 22.61) and alanine aminotransferase (ALT) (60.30 vs 30.85), compared with the C group (*P* < 0.05; Fig. 2D and E). Supplementation with *B. fragilis* significantly increased ALT levels (103.9 vs 60.30; *P* = 0.0014), while having little influence on AST levels (57.09 vs 47.90; *P* = 0.3469), compared with the H group. With regard to glucose metabolism, in comparison to the C group, HFD-fed mice had a higher fasting blood glucose (FBG) level (6.900 vs 4.933) and that level was drastically higher in the HB group than that in the H group (8.700 vs 6.900) (*P* < 0.05, Fig. 2F).

The levels of TC, TG, LDL-C, ALT, and AST in the liver of mice given an HFD were significantly higher than those in the C group (*P* < 0.05; Fig. S2A through E). Surprisingly, compared with the H group, supplementation of *B. fragilis* dramatically raised the ALT level in the liver (41.58 vs 35.49; *P* = 0.0313; Fig. S2D). Although the levels of TG, TC, LDL, and AST in the liver of the HB group were higher than those of the H group, the differences were not statistically significant (Fig. S2A through C and E). Furthermore, the H group had higher levels of lipopolysaccharide (LPS) in the serum (17.89 vs 7.458) and liver (9.531 vs 4.473) than the C group (*P* < 0.001; Fig. S1C and S2F). *B. fragilis* significantly increased serum (22.31 vs 17.89) and liver (14.16 vs 9.531) LPS levels in comparison to the H group (*P* < 0.001; Fig. S1C and S2F). In general, the findings showed that *B. fragilis* exacerbated liver dysfunction, including the accumulation of lipids in the liver and disturbances in glucolipid metabolism.

### Commensal *B. fragilis* aggravated lipid accumulation and lipid metabolism in the liver in HFD-fed mice

**Fig 3:**
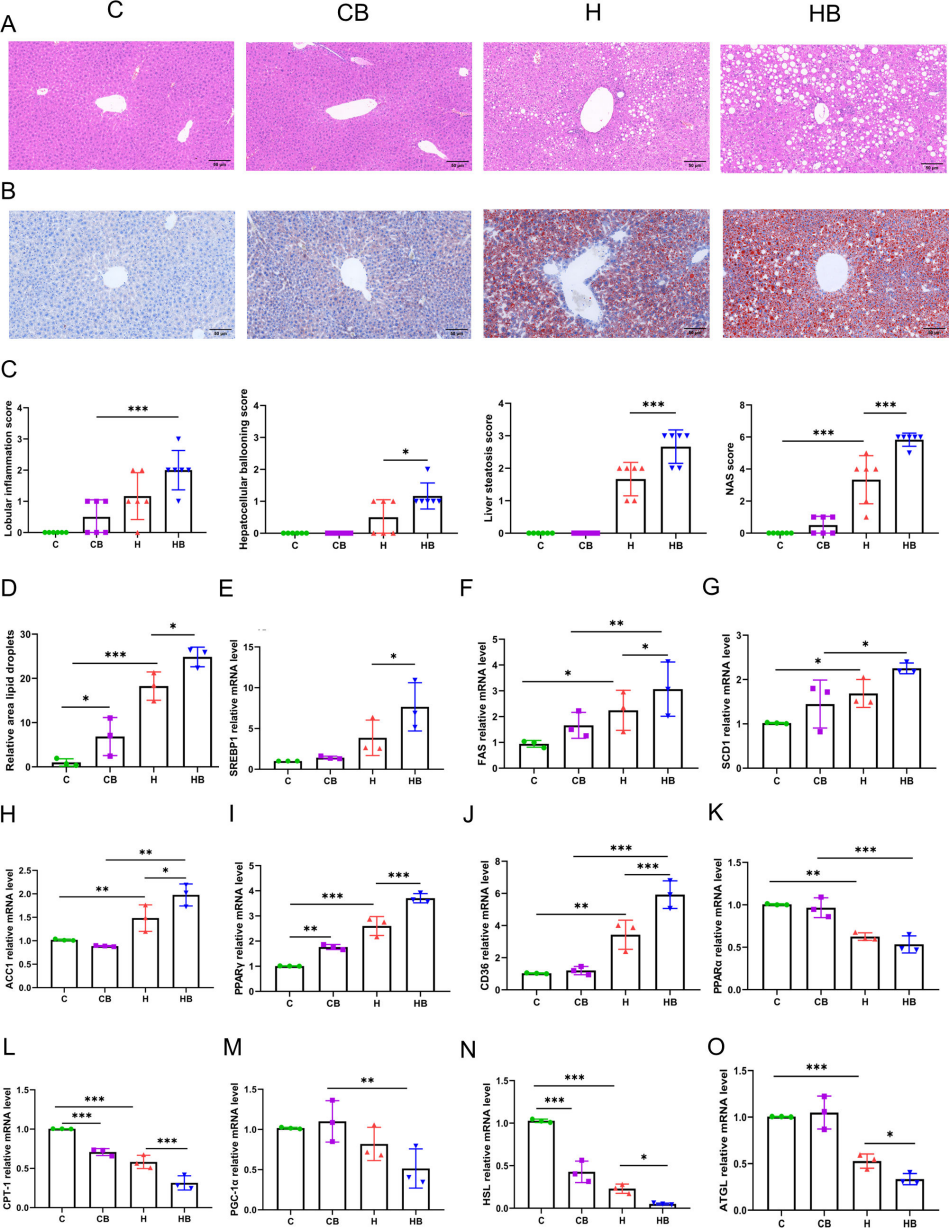
Commensal *B. fragilis* aggravated lipid accumulation and lipid metabolism in the liver in HFD-fed mice. (**A**) Representative HE staining of liver samples via microscope (200× magnification). (**B**) Representative ORO staining of liver samples via microscope (200× magnification). (**C**) NAS score. (**D**) Positive area rates in ORO staining. (**E**) SREBP1 mRNA levels. (**F**) FAS mRNA levels. (**G**) SCD1 mRNA levels. (**H**) ACC1 mRNA levels. (**I**) PPARγ mRNA levels. (**J**) CD36 mRNA levels. (**K**) PPARα mRNA levels. (**L**) CPT-1 mRNA levels. (**M**) PGC-1α mRNA levels. (**N**) HSL mRNA levels. (**O**) ATGL mRNA levels. Values were shown as the mean ± SD (*n*  =  6 in A and C, *n* = 3 in B and D-O). Statistical analysis involved a one-way ANOVA followed by Tukey’s multiple comparisons test or LSD test. *P < 0.05, **P < 0.01, and ***P < 0.001.

We subsequently conducted a histopathological examination of the liver. In hematoxylin-eosin (HE) staining, *B. fragilis* showed a significant increase in the occurrence of lipid droplets and hepatocyte ballooning degeneration as compared with the H group (Fig. 3A). According to the NAFLD activity score (NAS) histological system, the HB group demonstrated a significantly higher NAS compared with the H group (*P* < 0.05; Fig. 3C). Oil red O (ORO) staining demonstrated a higher presence of red-stained lipids in the H group, which was enhanced further in the HB group, as well as an increase in oil red-positive regions (Fig. 3B and D). HFD increased total TG and TC concentrations in the liver, as well as lipogenesis-related gene expression, including stearoyl coenzyme decarboxylase 1 (SCD1), sterol regulatory element binding protein 1 (SREBP1), fatty acid synthase (FAS), acetyl-CoA carboxylase 1 (ACC1), peroxisome proliferator-activated receptor γ (PPARγ), and fatty acid translocase (CD36), which were exacerbated by *B. fragilis* treatment (*P* < 0.05; Fig. 3F through J). Compared with the C group, the H group had no significant difference in sterol regulatory element binding protein 1 (SREBP1) levels, whereas an increase was observed in the HB group (*P* < 0.05; Fig. 3E). HFD downregulated fatty acid β-oxidation genes including peroxisome proliferator-activated receptor α (PPARα), carnitine palmitoyl transferase-1 (CPT-1), and peroxisome proliferator-activated receptor 1α (PGC-1α), and fatty acid decomposition genes including hormone-sensitive lipase (HSL) and adipose triglyceride lipase (ATGL) (*P* < 0.05; Fig. 3K through O). Compared with the H group, CPT-1, HSL, and ATGL were reduced by *B. fragilis* treatment (*P* < 0.05; Fig. 3L, N and O).

### Commensal *B. fragilis* altered gut microbiota in HFD-fed mice

**Fig 4:**
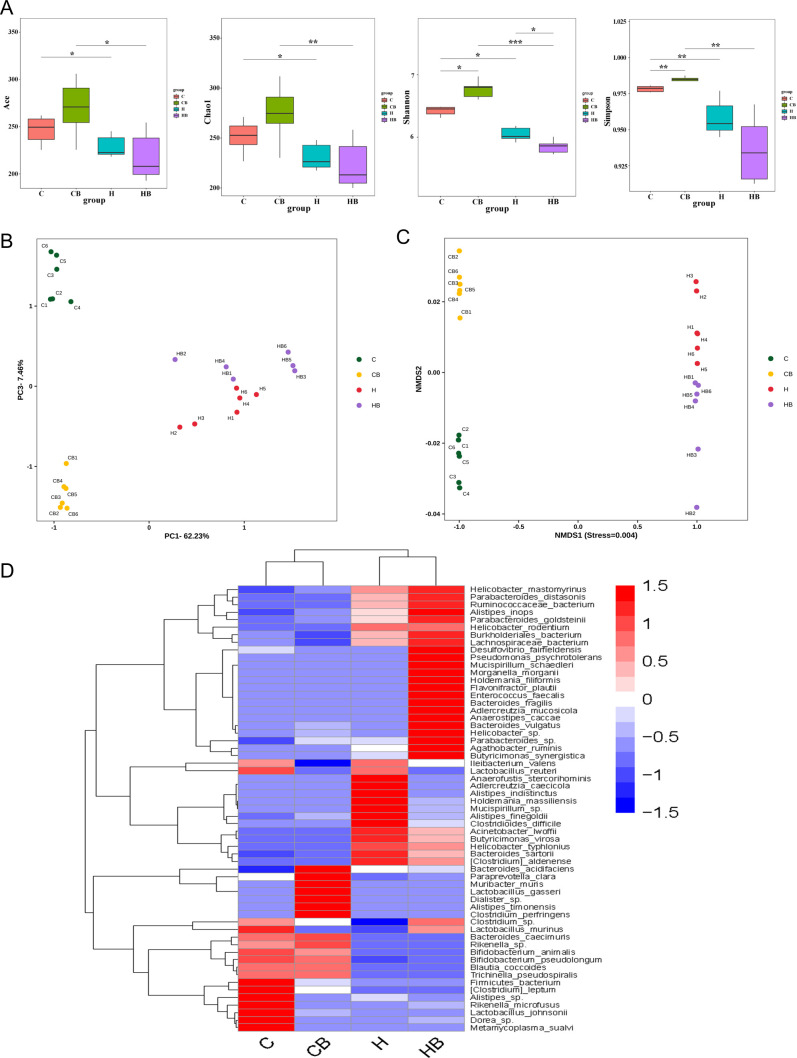
Commensal *B. fragilis* altered gut microbiota in HFD-fed mice. (**A**) Alpha diversity such as the ACE, Chao1, Shannon, and Simpson indexes for each group. (**B**) Beta diversity PCA. (**C**) NMDS score plot. (**D**) Heatmap of species clustering at the species level. Values were shown as the mean ± SD (*n* = 6). Statistical analysis involved the Kruskal-Wallis test and permutational multivariate analysis of variance (PERMANOVA) for beta diversity analyses. *P < 0.05, **P < 0.01, and ***P < 0.001.

Gut microbiota might have a role in the development of NAFLD. We investigated the impacts of commensal *B. fragilis* on gut microbiota by applying high-throughput sequencing of the 16S rRNA gene. All rarefaction curves approached a plateau, suggesting that the sequencing depth was adequate for subsequent analysis (Fig. S3A). Alpha diversity including the ACE index (*P* = 0.045), Chao1 index (*P* = 0.037), Shannon index (*P* = 0.0091), and Simpson index (*P* = 0.011) in the H group were substantially less than that in the C group (Fig. 4A). These findings demonstrated that the richness and diversity of the gut microbiota were substantially different from the C group to the H group. With the *B. fragilis* supplement, the Shannon index was significantly decreased (*P* = 0.046), while the ACE index (*P* = 0.39), Chao1 index (*P* = 0.51), and Simpson index (*P* = 0.075) were decreased with no significant difference, compared with the H to the HB groups (Fig. 4A). Beta diversity was used to measure the variability of the intestinal microbiota structure, and samples were characterized using principal component analysis (PCA) and non-metric multidimensional scaling (NMDS) to explore differences in the intestinal microbiota community composition. In the PCA and NMDS analyses, the samples showed distinct clustering patterns among the four groups (Fig. 4B and C). An HFD strongly influenced the community composition of the gut microbiota. Meanwhile, *B. fragilis* treatment showed an extensive effect on the diversity of bacteria.

In order to assess the compositional changes in the gut microbiota among the four groups, we carried out a systematic analysis at different levels of structures. At the phylum level, *Bacteroidota*, *Firmicutes*, and *Desulfobacterota* were identified as the top three most abundant microbial taxa (Fig S3B). The results showed that the relative abundances of *Bacteroidota* (62.68%) in the C group proved to be higher compared with those in the H group as well as the HB group (31.51% and 28.79%, respectively), while the C group exhibited a lower relative abundance of Firmicutes (32.46%) and Desulfobacterota (1.54%) compared with the H group (41.40% and 16.14%), respectively. Significantly, *B. fragilis* increased the relative abundance of *Desulfobacterota* (20.01%) (Fig S3B). These indicated that HFD and *B. fragilis* could change the gut microbiota community structure in mice. At the family level, *Muribaculaceae*, *Lachnospiraceae*, and *Desulfovibrionaceae* were the top three most abundant microorganisms. Compared with the H group (20.00% and 20.40%, respectively) and HB group (17.35% and 17.10%, respectively), the relative abundances of *Muribaculaceae* (51.89%) and *Lachnospiraceae* (24.40%) were higher in the C group. Similar to *Desulfobacterota*, *Desulfovibrionaceae* had a lower relative abundance in the C group (1.54%) in comparison with the H group (16.14%) and the HB group (20.01%) (Fig. S3C). At the species level, compared with the C group, the relative abundances of *Bacteroides_acidifaciens* (13.45%), *Paraprevotella_clara* (2.56%), *Muribacter_muris* (0.05%), *Lactobacillus_gasseri* (0.02%), *Dialister_sp* (0.05%), *Alistipes_timonensis* (0.03%), and *Clostridium_perfringens* (0.04%) were increased substantially in the CB group, while the relative abundances of *Firmicutes_bacterium* (3.58%), *Alistipes_sp* (0.28%), *Rikenella_microfusus* (0.20%), *Lactobacillus_johnsonii* (0.62%), *Dorea_sp* (0.27%), and *Metamycoplasma_sualvi* (0.02%) were significantly decreased in the CB group. The H group mice were noted at the species level, including higher *Anaerofustis_stercorihominis* (0.02%), *Adlercreutzia_caecicola* (0.02%), *Alistipes_indistinctus* (0.02%), *Holdemania_massiliensis* (0.67%), *Mucispirillum_sp* (1.11%), *Alistipes_finegoldii* (4.68%), and *Clostridioides_difficile* (2.01%) correlated to the C group. *B. fragilis* significantly increased the relative abundance of *Desulfovibrio_fairfieldensis* (0.28%), *Adlercreutzia_mucosicola* (0.04%), *Agathobacter_ruminis* (0.04%), *Bacteroides_fragilis* (4.81%), *Bacteroides_vulgatus* (1.68%), *Butyricimonas_synergistica* (0.64%), *Enterococcus_faecalis* (0.11%), *Flavonifractor_plautii* (0.13%), *Helicobacter_sp* (1.21%), *Holdemania_filiformis* (0.21%), *Morganella_morganii* (0.07%), *Mucispirillum_schaedleri* (0.56%), and *Parabacteroides_sp* (0.27%) in HFD mice (Fig. 4D).

To identify the distinct bacterial groups within various categories, we used the linear discriminant analysis effect size (LEfSe) technique (Fig S3D). Surprisingly, the findings revealed a distinct difference in gut microbiota in the four groups. The H group showed a higher abundance of *Firmicutes* within the phylum level, *Clostridia* within the class level, and *Oscillospirales* within the order level compared with the C group, whereas the CB group showed an increased prevalence of *Bacteroidales* within the order level. Similarly, *B. fragilis* changed gut microbiota, especially enriched in *Desulfovibrionales* in HFD mice (Fig. S3D). All of these results indicated that commensal *B. fragilis* changed gut microbiota.

### Effect of commensal *B. fragilis* on SCFAs of microbial metabolites

**Fig 5:**
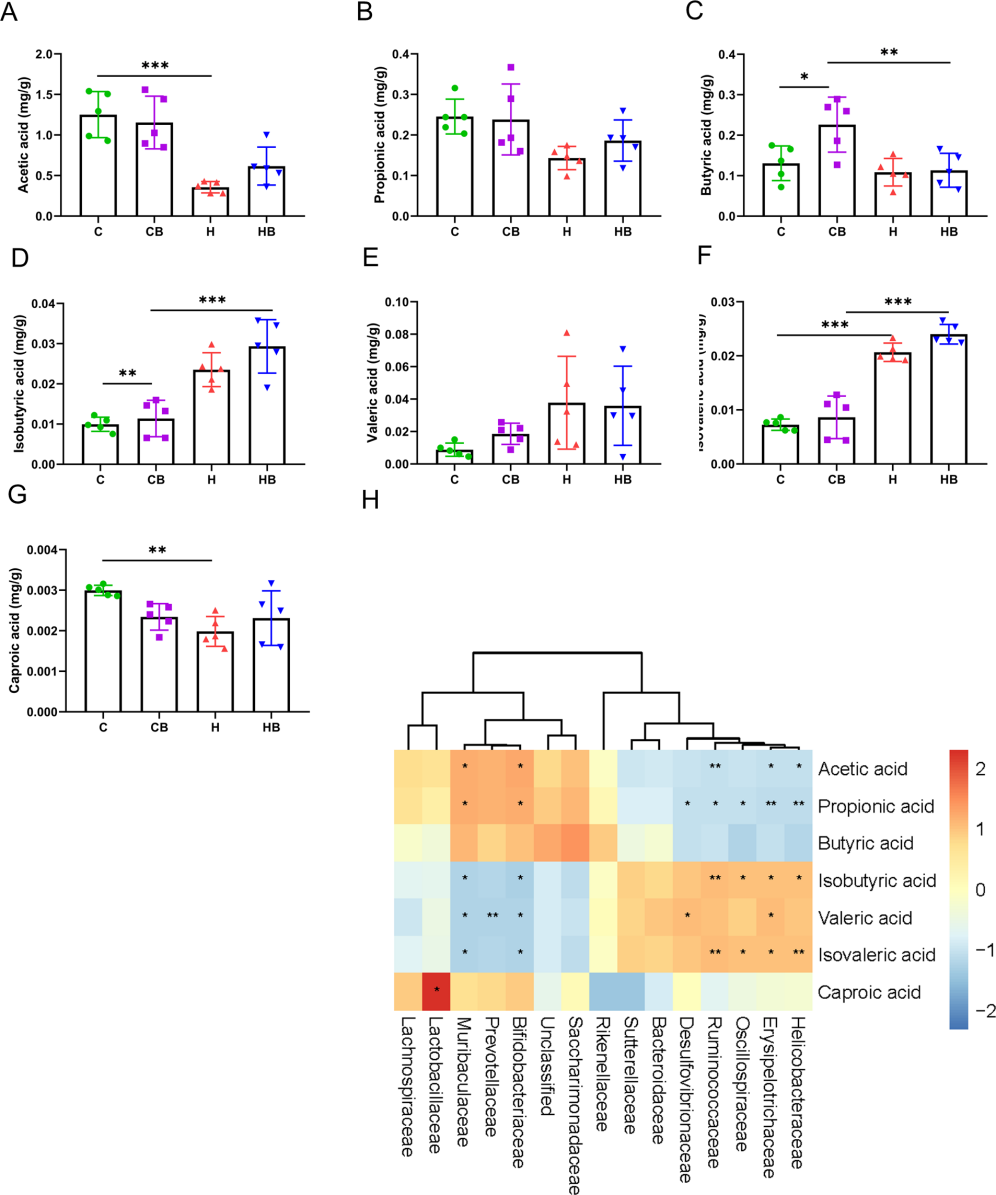
Effect of commensal *B. fragilis* on SCFAs of microbial metabolites. (**A–G**) SCFA levels. (**H**) Pearson correlation analysis was used to evaluate the correlation between the levels of metabolites and the prevalence of specific bacterial species. Values were shown as the mean ± SD (*n* = 5). Statistical analysis involved one-way ANOVA followed by Tukey’s multiple comparisons test. *P < 0.05, **P < 0.01, and ***P < 0.001.

To examine the variations in SCFA levels associated with gut microbiota in mice, we used gas chromatography-mass spectrometry (GC-MS) to analyze the levels of seven SCFAs found in mouse fecal samples: acetic acid (AA), propionic acid (PA), butyric acid (BA), isobutyric acid (IBA), valeric acid (VA), isovaleric acid (IVA), and caproic acid (CA). We observed comparable overall levels of SCFAs in the stool samples from all four groups, predominantly comprising AA, PA, and BA. AA was the most multiple of the three SCFAs, next to PA and BA. Following that, the SCFAs present in the stool samples were subjected to analysis. IBA and IVA levels were considerably higher in the H group compared with the C group, but AA and CA levels were significantly lower. The commensal *B. fragilis* increased BA and VA levels compared with the C group. Although the levels of AA, PA, BA, IBA, IVA, and CA were increased with commensal *B. fragilis* treatment compared with the H group, significant differences were not found (Fig. 5A through G). These results suggested that an HFD has a significant regulatory effect on SCFAs, which were metabolites of gut microbiota. *B. fragilis* had a regulatory effect on some SCFAs under a normal diet but had little effect on SCFAs under an HFD.

### Correlation analysis of intestinal microbiota with SCFAs, serum biochemistry, and liver lipid metabolism

To investigate the potential association between alterations in gut microbiota and SCFAs, Pearson correlation analysis was performed to assess the correlation between different bacteria and SCFAs (Fig. 5H). Microbial abundance and SCFA content of correlation analysis showed that *Bifidobacteriaceae* and *Muribaculaceae* had significantly negative correlations with AA. *Bifidobacteriaceae* and *Muribaculaceae* had clear positive correlations with PA, while *Helicobacteraceae*, *Erysipelotrichaceae*, *Oscillospiraceae*, *Ruminococcaceae*, and *Desulfovibrionaceae* had clear negative correlations with PA. Interestingly, there was no obvious correlation between BA and gut microbiota in this study. *Lactobacillaceae* had clear positive correlations with CA.

The correlation between serum metabolism and gut microbiota was further analyzed (Fig. S4A). The Pearson correlation analysis showed that *Desulfovibrionaceae* had clear positive correlations with TC, LDL-C, AST, and LPS levels in the serum. *Erysipelotrichaceae* had clear positive correlations with LDL-C levels in the serum. In addition, *Prevotellaceae* had an extremely negative correlation with serum levels of TG, TC, LDL-C, AST, and LPS. *Muribaculaceae* had an extremely negative correlation with serum levels of TC, LDL-C, and LPS. *Bifidobacteriaceae* had an extremely negative correlation with serum levels of LDL-C, AST, and LPS.

We then examined the correlation between gut microbiota and liver metabolism (Fig. S4B). The Pearson correlation analysis showed that *Bacteroidaceae* had clear positive correlations with TC, LDL-C, AST, and LPS levels in the liver. *Desulfovibrionaceae* had clear positive correlations with the liver level of TG. *Prevotellaceae* had clear negative correlations with TG TC, ALT, AST, and LPS levels in the liver. *Muribaculaceae* had clear negative correlations with the levels of TC in the liver. Among the genes involved in liver lipid metabolism, Pearson’s correlation analysis showed that ATGL had clear positive correlations with *Bifidobacteriaceae*, *Prevotellaceae*, and *Muribaculaceae*, while having clear negative correlations with *Erysipelotrichaceae* and *Desulfovibrionaceae*. HSL had clear negative correlations with *Erysipelotrichaceae*. ACC1 had clear positive correlations with *Desulfovibrionaceae*. CD36 had a clear positive correlations with *Desulfovibrionaceae*, while had a clear negative correlation with *Prevotellaceae*.

## DISCUSSION

An increasing amount of evidence demonstrate that intestinal dysfunction has a profound impact on a variety of phenotypic alterations and the development of disorders relevant to an HFD, including NAFLD (4, 5, 22). The commensal microbiome has been demonstrated to be a significant contributor to gastrointestinal and liver disorders (23). A viable mouse model was established in this study by introducing a single microbe into a diverse microbial community to research the impact of commensal *B. fragilis* on the metabolism and interactions between microbes under an HFD condition. The findings indicated that the disorder of lipid metabolism induced by an HFD was worsened by the presence of commensal *B. fragilis*. This ultimately exacerbated the development and progression of NAFLD in a murine model.

After a 16-week duration on an HFD, the mice in the H group showed a substantial rise in body weight compared with those in the C group. This finding complied with previous studies that reported significant weight gains in male C57BL/6J mice after being fed an HFD for a duration of 12 weeks (24, 25). The presence of *B. fragilis* symbiont in conjunction with a diet rich in fats resulted in significant weight gain and enhanced lipid synthesis in adipose and hepatic tissues. Additionally, adipose tissue accumulation was observed in HB mice along with a rise in serum levels of TC and TG, as showed in a previous study (18). It is interesting that *B. fragilis* alone in chow diet increased body weight, lipid accumulation in the liver, and lipogenesis-related gene expression in our study. Consistent with a previous animal study, *B. fragilis* colonization could increase the weight of the liver, muscle, and epididymal fat pad (EPF) in *B. fragilis* gnotobiotic mice (26). A clinical study found that an increased abundance of *B. fragilis* was associated with obesity in peri- and post-menopausal women (20). Another clinical study showed a higher *B. fragilis* in infants born vaginally to overweight mothers (27). All of these studies suggested that *B. fragilis* may be an important therapeutic target for obesity intervention. In the future, more research will be desired to determine the precise mechanism. Lipogenesis, fatty acid absorption, and fatty acid oxidation constitute just some of the physiological processes that constitute the complex and variable liver lipid metabolism (28). HFD aggravated the expression level of hepatic lipid metabolism genes, and we first found *B. fragilis* further aggravated hepatic lipid metabolism. In our study, HFD mice initiated with *B. fragilis* showed higher serum and liver levels of ALT. According to a new study, *B. fragilis* had a significant association with TBA, ALT, and AST in intrahepatic cholestasis of pregnancy (29).

In our study, supplementation of *B. fragilis* increased FBG levels and exacerbated glucose metabolism in HFD-fed mice. Supplementation of *B. fragilis* in mice with increased intestinal permeability led to an expedited progression of the disease and a swift onset of hyperglycemia (19). NAFLD and NASH are associated with increased permeability of the intestinal barrier and translocation of bacteria or bacterial products into the circulation (30, 31). HFD-induced dysbiosis may result in bacteria being able to cross the impaired gut vascular barrier. Bacterial translocation during HFD may induce persistent inflammation, which, coupled with high lipid content in the diet, may drive metabolic disorders (30, 32).

*B. fragilis* is a crucial element of the commensal microbiota found in the human colon (19). In our study, no significant difference was observed in diversity index (Simpson) and richness index (Chao and ACE) between the H and HB groups. Similar to previous studies, the effect of *B. fragilis* on the diversity and richness of the microbiota may vary depending on whether the sample is mucosal or fecal and may also be related to the number of samples (33, 34). The beta diversity analysis revealed a significant change in the PCA index, indicating that although *B. fragilis* did not change the type of bacterial taxa present, it changed the relative abundance of bacteria at different taxonomic levels. As a result, the structure of the gut flora changes. Intestinal biological interactions between total are dynamic and complex. Compared with the C group, the H group exhibited a reduction in alpha diversity and an increase in *Firmicutes*/*Bacteroidetes* ratio, consistent with previous research (35–37). *Desulfobacterota* and *Desulfovibrionaceae* significantly increased in HFD mice, which is consistent with previous research (38, 39). *B. fragilis* further increased their levels with HFD. *Desulfovibrionaceae* are considered to be potentially harmful bacteria in the gut (40). HFD-induced lipopolysaccharide-associated bacteria in mice included *Firmicutes*, *Proteobacteria*, and Desulfovibrionaceae (41). In our study, levels of LPS in the serum and liver in the H and HB groups increased, which may be attributed to an increase in harmful bacteria associated with LPS in the gut.

SCFAs are the essential metabolites obtained from microbial digestion of carbohydrates and play an essential part in metabolism, neurological system, and immune function homeostasis (42). Similar to previous studies, an HFD significantly reduced the levels of AA, PA, and BA although there was no significant difference (43, 44). Interestingly, supplementation with commensal *B. fragilis* resulted in changes in the gut microbiota, but there was no significant effect of commensal *B. fragilis* on fecal SCFAs. Different from previous studies, Pearson correlation analysis showed that bacteria that produce SCFAs, such as *Lachnospiraceae* and *Rikenellaceae*, had no significant correlation with SCFA production (45). One probable reason is that bacteria create SCFAs that have an associated positive effect with some SCFA levels and an adverse relationship with others, causing increases in SCFA levels to be reversed. Meanwhile, these findings propose alternative mechanisms that may contribute to the compromised glucose metabolism and heightened lipid metabolism observed in HB mice.

Previous studies associated *Desulfovibrionaceae* with diabetes, overweight, NAFLD, and other metabolic diseases (46–48). There was an obviously positive correlation between *Desulfovibrionaceae* and the levels of TC, LDL-C, and AST in both serum and liver, indicating that a possible reason for *B. fragilis* to aggravate fatty liver is caused by the change of intestinal flora and the increase of harmful bacteria *Desulfovibrionaceae*. In addition, *Erysipelotrichaceae* is also associated with fatty liver (49, 50). *Erysipelotrichaceae* exhibited a significant positive association with bloodstream TC and liver TG levels, as well as a substantial positive correlation with CD36 and ACC1, but a substantial negative correlation with ATGL. Under an HFD, the increase of harmful bacteria aggravated the abnormal lipid metabolism in the liver.

In summary, *B. fragilis* exacerbates HFD-induced overweight, glucose dysregulation, and lipid metabolic dysregulation under long-term HFD feeding conditions. With an HFD, commensal *B. fragilis* changes the gut microbiota and increased translocation of bacterial-derived substances such as LPS may result in altered glucose homeostasis and lipid metabolism. *B. fragilis* strain is a member of the intestinal tract, under the background of HFD intervention effects on the metabolism of the host. This study demonstrates the essence in studying the importance of specific gut symbionts in the development of obesity and NAFLD, in order to develop strategies for controlling these symbionts and improving metabolic outcomes.

## MATERIALS AND METHODS

### Culture of *B. fragilis*

*B. fragilis* (ATCC 25285) was derived from the China Center for Type Culture Collection. *B. fragilis* was cultured on Columbia blood agar at 37°C with anaerobic conditions for 24**–**48 hours. Subsequently, *B. fragilis* was cultivated in a brain heart infusion medium at 37°C with anaerobic circumstances for 24–48 hours. All mediums were purchased from Qingdao Hope Bio-Technology Company (Qingdao, China). *B. fragilis* was harvested and resuspended in PBS for measurement of optical density (OD) at 600 nm, as described previously (15).

### Animals

Six-week-old C57BL/6J inbred specific pathogen-free (SPF) male mice were acquired from Hunan Slake Jingda Experimental Animal Co. Ltd. (Changsha, China). Mice were housed in a regulated environment, providing unrestricted availability of food and water, under a 12-hour daylight cycle at 25°C ± 2°C. Mice were assigned at random into four groups after a week of adaptation (C, CB, H, and HB group, *n* = 8). The mice in the C and CB groups were provided with a standard chow diet (Jiangsu Xietong), while the mice in the H and HB groups were provided with an HFD (Jiangsu Xietong) for a duration of 16 weeks. Mice in CB and HB groups were administered intragastrically with *B. fragilis* at an oral dosage of 2 × 10^8^ CFU per 200 µL every alternate day for the last 8 weeks (51). Mice in the C and H groups were administered with the equivalent amount of PBS. All animals have been cared for in compliance with the guidelines of the Institutional Animal Care and Use Committee at Huazhong University of Science and Technology (IACUC Number: 3326).

### Specimen collection

The total weight of the mice was measured weekly during the course of the experiment. In the last week before euthanasia, the FBG levels were assessed following an 8-hour period of fasting. The mice were subjected to tail vein blood collection. An adaptable blood glucose meter and blood glucose test sheets were utilized to measure blood glucose levels. The excrement of mice was freshly gathered and preserved in a refrigerator at −80°C. The mice were starved overnight, weighed, and euthanized under anesthesia in accordance with guidelines for experimental animals. Blood was taken and allowed to coagulate for 30 minutes in microcentrifuge tubes. The upper serum was centrifuged next, and then, it was kept for further examination at −80°C. Weigh liver, kidneys, epididymal fat, and perirenal fat weight after euthanasia. The tissue samples were washed with pre-cooled saline solution to eliminate any residual blood or impurities adhering to the surface of the tissue and preserved in a refrigerator at −80°C.

### Biochemical assays in serum and liver

The tissue blocks were weighed, cut into pieces, and homogenized by ultrasound in a proportion of 1:9 (tissue weight to PBS volume). The homogenate was transferred to a centrifuge tube and centrifuged for 10 minutes at 4°C at 1,000 *g*. To prevent recurrent cycles of freezing and thawing, the supernatant was gathered and stored at −80°C. Assay reagents (Nanjing Jiancheng Bioengineering Institute, China) were used to detect serum and liver levels of TG, TC, AST, ALT, LDL-C, and HDL-C in accordance with the manufacturer’s directions (52).

### RT-qPCR analysis

The RNA-Easy Isolation Reagent was used for extracting total RNA (Vazyme, China). The cDNA was then reverse transcribed using HiScript II QRT SuperMix for qPCR (Vazyme, China), according to the manufacturer’s directions (53). The mRNA levels of SREBP1, FAS, SCD1, ACC1, PPARγ, ACC1, CD36, PPARα, CPT-1, PGC1α, HSL, and ATGL were measured. The primers were presented in Table 1.

**TABLE 1:** Primer sequences

Gene	Forward primer (5′−3′)	Reverse primer (5′−3′)
SREBP1	GTGGAGACGCTTACCCCTC	CCAAAGGATTGCAGGTCAGAC
FAS	AGTTTAAAGCTGAGGAGGCGGGTT	CAGGTTGGCATGGTTGACAGCAAA
SCD1	CCGGAGACCCCTTAGATCGA	TAGCCTGTAAAAGATTTCTGCAAAC
ACC1	AATGAGACTAGCAAAACAATC	ACGACCAAACAAAGAAATA
PPAR-γ	TGGAGCCTAAGTTTGAGTT	AGCAGGTTGTCTTGGATGT
CD36	AGAACTCTTGTGGGGTTAC	GGACTCAATTATGGCAACT
PPAR-α	AGCAACAACCCGCCTTTT	GCACTGGCAGCAGTGGAA
CPT-1	GTGTCCAAGTATCTGGCAGTC	ATAGCCGTCATCAGCAACC
PGC-1α	TCATCACCTACCGTTACAC	ACAGCTCGAAGTCAGTTTC
HSL	TTCGGGGAGCACTACAAAC	CCACGCAACTCTGGGTCTA
ATGL	GACCTGATGACCACCCTTTC	TGCTACCCGTCTGCTCTTT
GAPDH	TCAACGGCACAGTCAAGG	TTAGTGGGGTCTCGCTCC

### HE staining

The hepatic tissues were fixed in a neutral fixative containing 4% paraformaldehyde for a minimum of 24 hours. After undergoing dehydration and transparency processes, the tissue was embedded in wax to form a block. Thin sections measuring 4 μm were then dewaxed, hydrated, stained, dehydrated again, and covered with a coverslip. An optical microscope (Olympus) was utilized for the observation and photography of the sections. The liver histological score was determined by using the NASs. The scoring system contains steatosis (0–3), lobular inflammation (0–2), hepatocellular ballooning (0–2), and fibrosis (0–4). Liver fibrosis was not assessed during the limited duration of the modeling period, as described previously (54).

### Liver staining with ORO

To identify liver lipid droplet accumulation, an ORO kit (Servicebio, Wuhan, China) was used. With a frozen slicer, liver tissue sections were made at a thickness of 10 µm and dyed according to the directions of the ORO staining kit. Lipid droplets emerged as red-stained structures, while nuclei were dark-blue stained. ImageJ software was used to quantify the lipid droplets.

### The enzyme-linked immunosorbent assay

LPS concentrations among liver tissues and serum were measured using an enzyme-linked immunosorbent test (Bioswamp, China), as directed by the manufacturer.

### 16S rRNA gene sequencing

The genomic DNA of bacteria was isolated from frozen stool samples by using the CTAB/SDS technique, following the manufacturer’s guidelines (55). The Qubit assay was employed for quantifying DNA concentration, while using 1% agarose gel electrophoresis, the integrity of isolated genomic DNA was assessed. Primer set 341F-806R was used to amplify the 16S rRNA V3/V4 region. 341F (5′-CCTAYGGGRBGCASCAG-3′) was the forward primer and 806R (5′-GGACTACHVGGGTWTCTAAT-3′) was the reverse primer. The amplified PCR products were mixed to build the library, and the sequencing adapter was added. After PCR amplification, the small fragments such as primer dimer were removed by magnetic bead sorting, and the library index information was recorded. Samples exhibiting a unique amplification outcome were chosen for subsequent investigation. After preparing DNA libraries, sequencing was conducted using an Illumina NovaSeq platform, following the standard protocols provided by Baiaoweifan Biotechnology Co. Ltd. (Wuhan, China). Amplicon sequencing data, primer removal, quality control, ASV inference, double-end splicing, and ASV feature table generation for further analysis are the data analysis process. For quality control of the original sequencing sequence, use the FLASH software. Select a window size of 50 bps, and filter readings with a tail mass value less than 20. Based on the overlap connection between PE reads, the paired reads are combined into a sequence with a minimum overlap length of 10 bp. There is no match between the screening and the sequence, and the maximum permitted mismatch ratio in the concatenated sequence’s overlap area is 0.2. Utilizing the barcode and the primers at the start and finish of the sequence, distinguish the sample; if required, change the direction of the sequence. A maximum of two primer mismatches are allowed, while zero mismatches are allowed for the barcode. Using default parameters, the DADA2 method in the Qiime2 process is employed to denoise and optimize the sequence after quality control stitching. The sequences obtained after DADA2 denoising are commonly referred to as ASVs. The number of denoised sequences for each sample was normalized to 4,000 in order to reduce the effect of sequencing depth on the outcomes of following analyses, such as alpha diversity and beta diversity. Even after flattening each sample, the average coverage of Good’s may still be 97.90%. Based on the Sliva 16S rRNA database, a Naive Bayes classifier from Qiime2 was used to conduct the taxonomic analysis of ASVs.

### Determination of fecal SCFAs

GC-MS was used to measure the amounts of SCFAs in stool samples. As in the prior investigation, samples were pretreated (56). The supernatant was immediately subjected to GC-MS analysis after being passed through a 0.22-µm syringe filter. The conditions for GC analysis were as follows: the capillary column size was 30 mm × 0.25 mm × 0.25 µm. The carrier gas, helium, had a flow rate of 1.2 mL/min. The temperature of the front injection was 200°C. The oven temperature ramp was as follows: 90°C for 1 min, then 100°C at a rate of 25°C/min, then 150°C at a rate of 20°C/min, held for 0.6 min, and 200°C at a rate of 25°C/min, maintained for 0.5 min, after operating for 3 min. The transfer line temperature was 230°C, the ion source temperature was 230°C, and the quad temperature was 150°C. An electron bombardment ionization (EI) source with full scan and sim scanning modes at an electron energy of 70 eV was used in the mass spectrometry analysis. An Agilent chemical workstation was used to process all of the data. Based on the standard, the MWDB (Metware Database) was created to qualitatively interpret mass spectrometry data.

### Statistical analysis

GraphPad Prism 8.0 and SPSS 22.0 statistical software were used for data analysis, with assessment of normality (Kolmogorov-Smirnov test) and variance homogeneity (Fisher’s F-test). One-way or two-way ANOVA was employed to compare data across multiple groups, followed by Tukey’s multiple comparisons test or LSD test. Mean ± SD values were reported. Statistical tests included Kruskal-Wallis Pairwise test for alpha diversity analysis, PERMANOVA for beta diversity analyses, and non-parametric Pearson test to examine the correlation between gut microbiota and host metabolism.

## Data Availability

The raw sequencing data were submitted to the National Center for Biotechnology Information (NCBI) database and given the accession number PRJNA1013139.

## SUPPLEMENTAL MATERIAL

Supplemental figures spectrum.03393-23-s0001.docx Fig. S1 to S4.

ASM does not own the copyrights to Supplemental Material that may be linked to, or accessed through, an article. The authors have granted ASM a non-exclusive, world-wide license to publish the Supplemental Material files. Please contact the corresponding author directly for reuse.
