# Supplementary material for: Bacteroides fragilis aggravates high-fat diet-induced non-alcoholic fatty liver disease by regulating lipid metabolism and remodeling gut microbiota
Source: Microbiol Spectr. 2024 Feb 27;12(4):e03393-23. doi: 10.1128/spectrum.03393-23 (PMC10986510; doi:10.1128/spectrum.03393-23)
Supplement: Supplemental figures — Fig. S1 to S4. [file spectrum.03393-23-s0001.docx]

**Supplemental Information for:**

## Bacteroides fragilis Aggravates High-Fat Diet-Induced Non-alcoholic Fatty Liver Disease by Regulating Lipid Metabolism and Remodeling Gut Microbiota

**Yumei Huang,^a^ Jiali Cao****,^a^ Mengpei Zhu,^a^ Ziwen Wang,^a^ Ze Jin,^a^ and Zhifan Xiong^a^***

^a^Department of Gastroenterology, Liyuan Hospital, Tongji Medical College, Huazhong University of Science and Technology, Wuhan, Hubei, China

*Address correspondence to Zhifan Xiong: xiongzhifan@126.com

**Supplemental Figures**

**FIG S1** Commensal B. fragilis induced an increase in body weight and LPS level in HFD-fed mice

**FIG S2** Commensal B. fragilis aggravated lipid metabolism and liver dysfunction in HFD-fed mice

**FIG S3** Commensal B. fragilis altered gut microbiota in HFD-ed mice

**FIG S4** Correlation analysis of intestinal microbiota with serum biochemistry and liver lipid metabolism

**
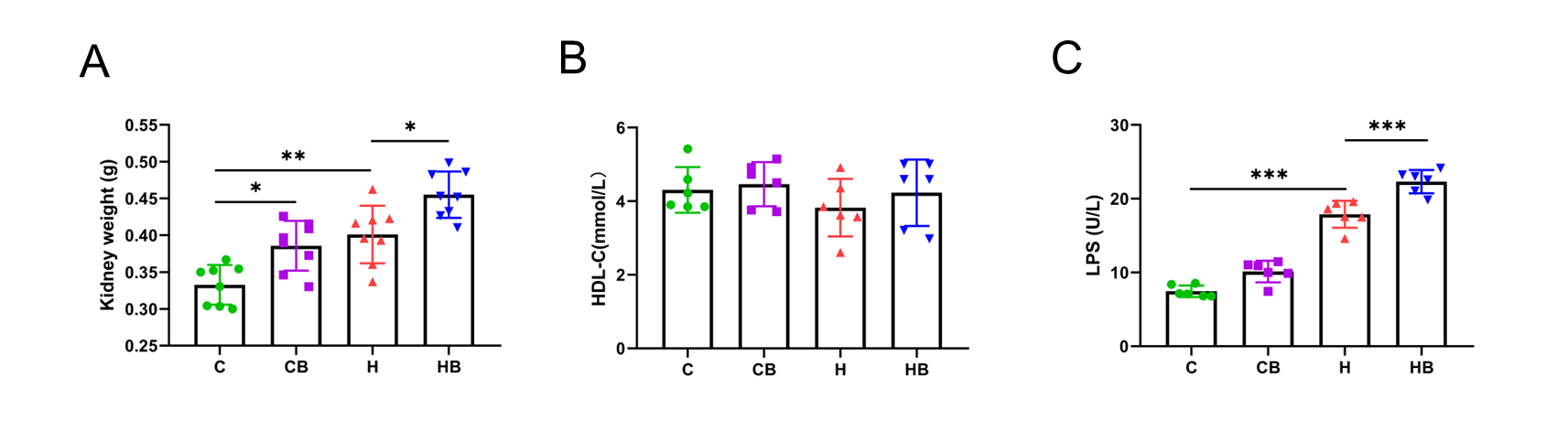
**

**FIG S1** Commensal B. fragilis induced an increase in body weight and LPS level in HFD-fed mice. (A) Kidney weight. (B) HDL-C levels in the serum. (C) LPS levels in the serum. Values were shown as the mean ± SD (n=8 in A, n=6 in B and C). Statistical analysis involved a one-way ANOVA followed by the Tukey's multiple comparisons test. * P < 0.05, ** P < 0.01, *** P < 0.001


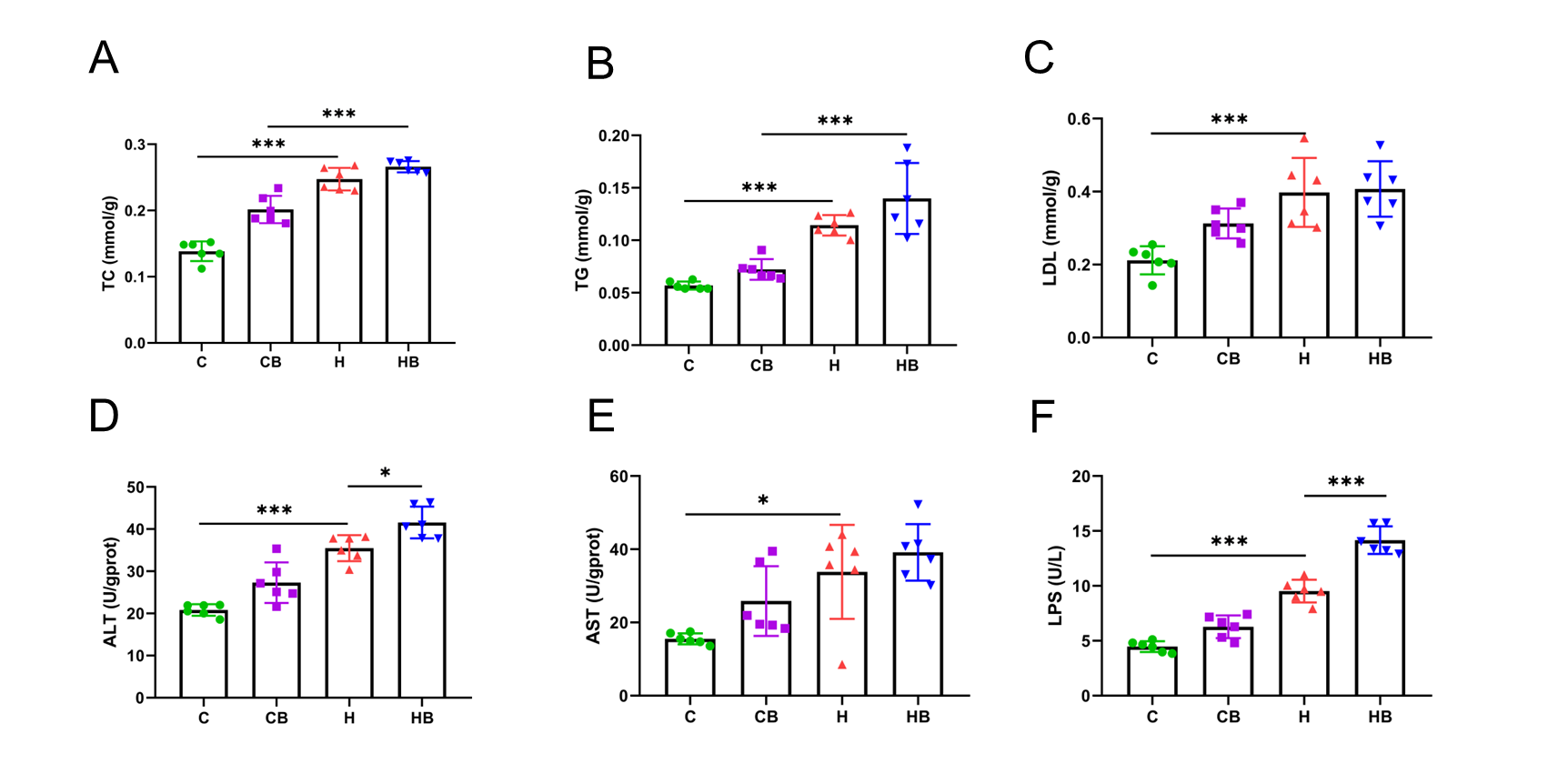


**FIG S2** Commensal B. fragilis aggravated lipid metabolism and liver dysfunction in HFD-fed mice. (A) TC levels in the liver. (B) TG levels in the liver. (C) LDL-C levels in the liver. (D) ALT levels in the liver. (E) AST levels in the liver. (F) LPS levels in the liver. Values were shown as the mean ± SD (n=6). Statistical analysis involved a one-way ANOVA followed by the Tukey's multiple comparisons test. * P < 0.05, ** P < 0.01, *** P < 0.001.


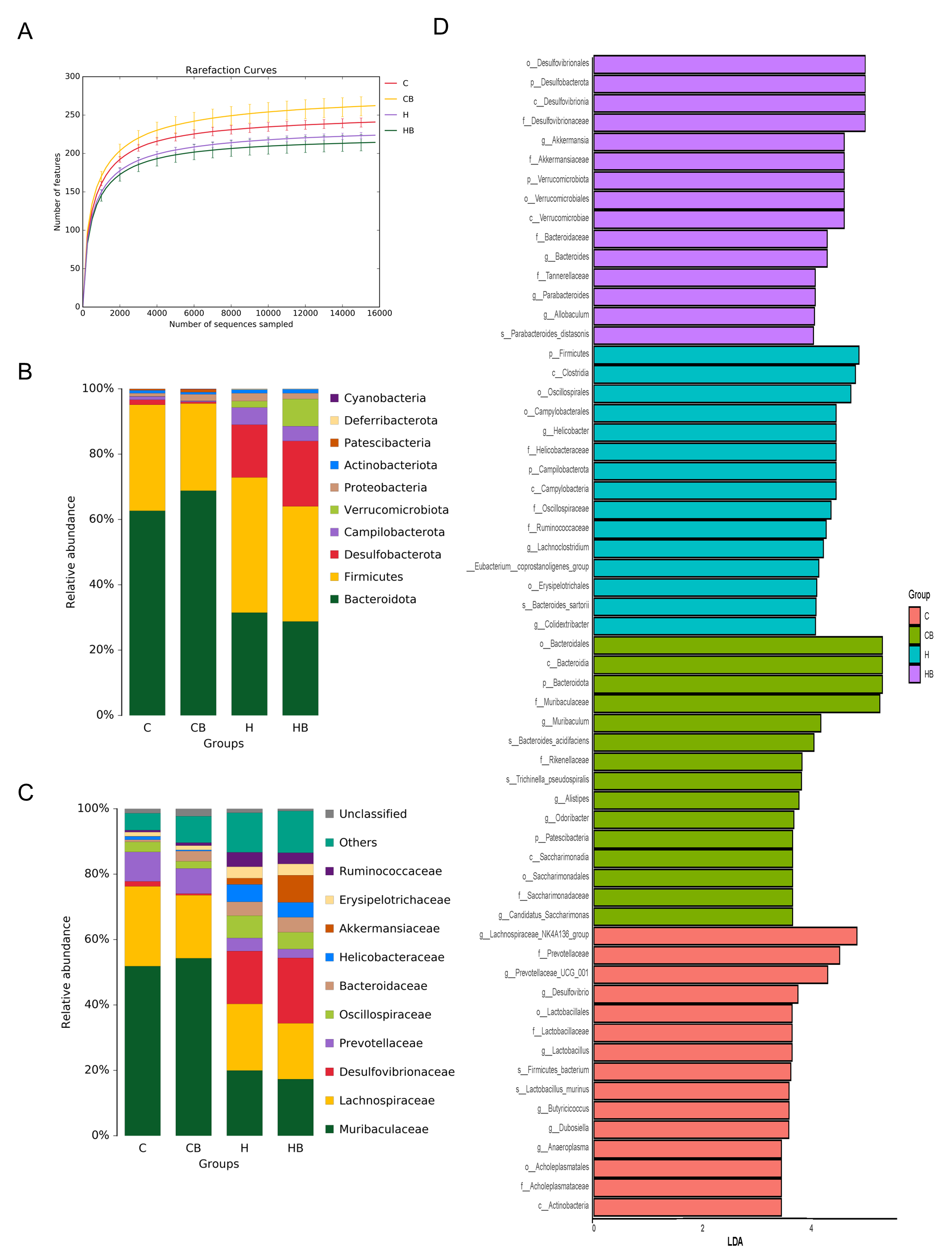


**FIG S3** Commensal B. fragilis altered gut microbiota in HFD-ed mice. (A) The rarefaction curves of all samples. (B) The top 10 kinds of bacteria ranked by phylum. (C) The top 10 kinds of bacteria by family. (D) Histogram of LEfSe analysis. The Kruskal-Wallis test was employed for statistical analysis. Differentially abundant taxa were analyzed using the LEfSe software, with a default LDA score filter value of 3.


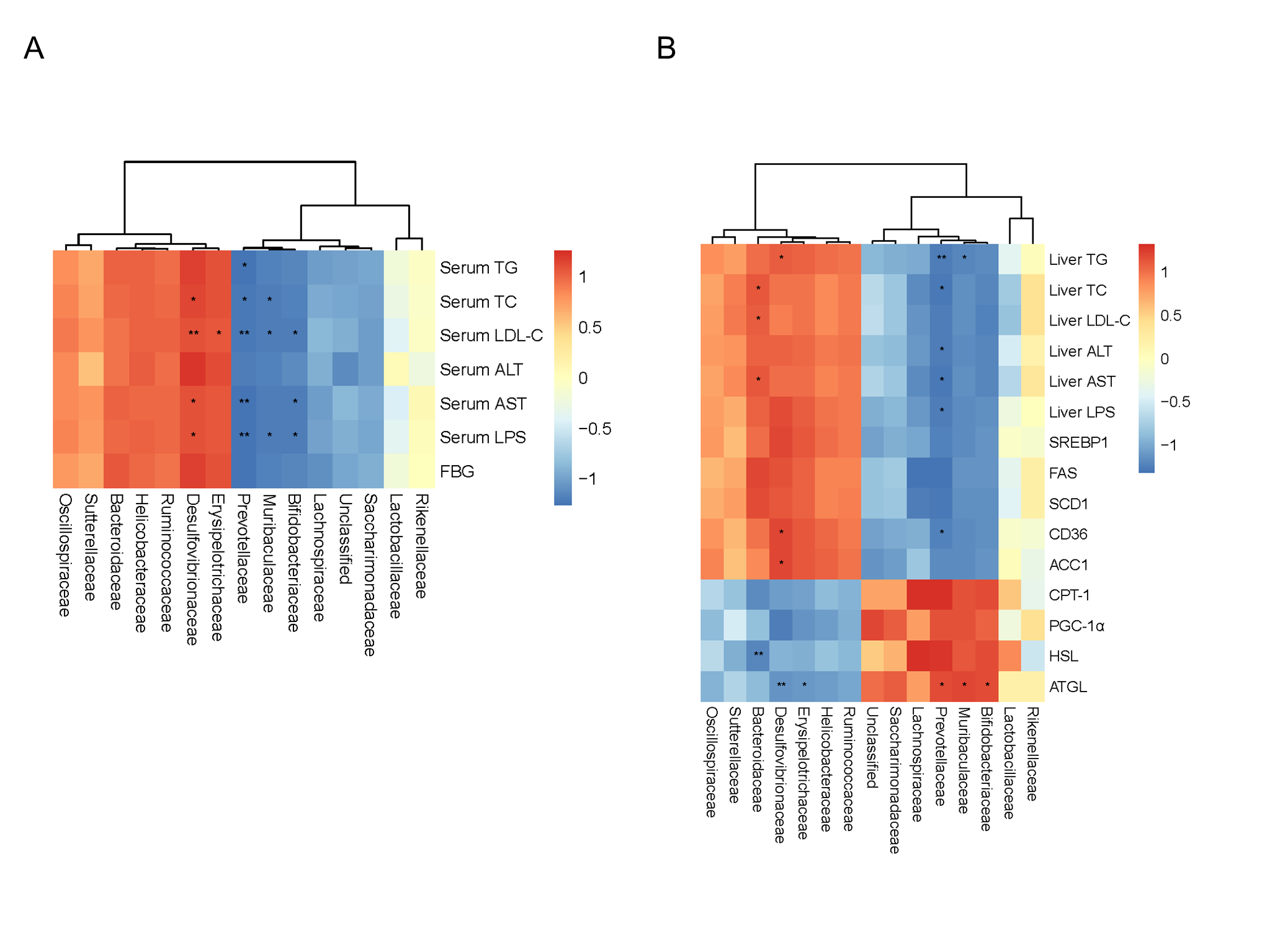


**FIG S4** Correlation analysis of intestinal microbiota with serum biochemistry and liver lipid metabolism. (A) Pearson correlations between the serum metabolism and gut microbiota. (B) Pearson correlations between the liver metabolism and gut microbiota. Red or blue color means positive or negative correlation. * P < 0.05, ** P < 0.01, *** P < 0.001.
